# Comparative Evaluation of Adiposity Indices as Predictors of Hypertension among Brazilian Adults

**DOI:** 10.1155/2018/8396570

**Published:** 2018-06-05

**Authors:** Maurílio T. Dutra, Diego B. V. Reis, Karla G. Martins, André B. Gadelha

**Affiliations:** ^1^Technical School of Health, State Department of Education of the Federal District, Brasília, Brazil; ^2^Federal Institute of Education, Science and Technology of Brasília, Brasília, Brazil; ^3^Mauá Institute of Research and Education, Vicente Pires, Federal District, Brazil

## Abstract

**Purpose:**

To compare several anthropometric indices in the prediction of hypertension among adults.

**Methods:**

This is a cross-sectional study. Five hundred and eighteen adult men and women (40.9 ± 10.5 years; 1.62 ± .09 m; 72.3 ± 15.6 kg) volunteered to participate and underwent blood pressure and anthropometric measures. Anthropometric assessments were used to calculate body mass index (BMI), waist circumference (WC), waist-to-hip ratio (WHR), waist-to-stature ratio (WSR), body adiposity index (BAI), and conicity index (C). Comparisons between men and women were carried out by independent* t*-test and chi-square test. Cut-off points for each adiposity index to predict hypertension were obtained using Receiver Operating Characteristic (ROC) curve analyses. The significance level was set at* P* ≤ .05.

**Results:**

All adiposity indices regarding both genders showed significant odds ratios, except BAI (odds ratio: 1.534; CI: 0.916–2.571) for women. In men, WHR and WSR were considered as more balanced indices regarding their sensitivity (AUC: 73.8 and 71.4, respectively) and specificity (AUC: 77.6 and 73.1, respectively). In women, WHR and WSR presented areas under the ROC curves higher than C index (*P* = .007) and BAI (*P* = .03), respectively.

**Conclusion:**

Indices that consider abdominal adiposity such as WC, WHR, and WSR have a stronger relationship with hypertension compared to others.

## 1. Introduction

Hypertension is characterized by high and sustained values of blood pressure (BP). It is a chronic multifactorial condition often associated with functional and structural alterations in several organs. Also, hypertension is related to metabolic alterations with a consequent increase in the risk of fatal and nonfatal cardiovascular events [1]. As it is highly prevalent both in developed [2] and low-to-middle-income countries [3], hypertension has been considered a major public health issue worldwide [4]. Particularly, Brazil is a middle-income country that presents a high prevalence of hypertension, reaching 25% of the population [5].

Risk factors for hypertension include age, gender, ethnicity, socioeconomic status, and lifestyle-related factors, such as sodium intake and sedentary lifestyle [1]. Moreover, excess body weight caused by genetic and/or sedentarism and bad nutrition is commonly associated with the incidence of hypertension [1]. Noteworthy, several adiposity indices, such as body mass index (BMI), waist circumference (WC), conicity index (C), and waist-to-hip-ratio (WHR), have been extensively applied to assess cardiovascular risk factors among adults and elderly people [6–8]. In fact, studies have shown that those indices, particularly when WC is accounted for, are good predictors of hypertension [7, 8].

In addition, other adiposity indices that may be useful for predicting cardiovascular diseases have been proposed in the literature, such as the body adiposity index (BAI) [9] and the waist-to-stature ratio (WSR) [10]. Previous results indicate that BAI is a good and valid index for estimating body fat [9]. Meanwhile, WSR was found to be more strongly associated with cardiovascular risk factors (i.e., lipid profile and blood pressure) than BMI, WHR, and WC in a representative sample of Chinese people [10].

As anthropometric derived indices are cost-effective strategies to assess and manage health outcomes, it would be useful to analyze which of those indices has the best sensitivity and specificity to predict hypertension. Of note, middle-income countries like Brazil have been facing a growing incidence of obesity and hypertension [1, 11]. Thus, a comparative evaluation of traditional and newer adiposity indices in the prediction of hypertension among Brazilian people is warranted. Therefore, the aim of the present investigation was to analyze and compare several anthropometric indices in the prediction of hypertension among Brazilian adults.

## 2. Materials and Methods

### 2.1. Study Design

The present cross-sectional study was designed to analyze and compare several anthropometric indices in the prediction of hypertension among Brazilian adults. To reach this aim, adults of both genders underwent anthropometric evaluations in several locations of an urban community of the Federal District, Brazil. People were invited as they walked through the evaluation sites that had previously been set up by the researchers in distinct locations of the community. Participants answered a questionnaire addressing medical history, hypertension diagnosis, tobacco use, alcohol consumption, and physical activity habits. Body mass and height, as well as waist and hip circumferences, were measured to analyze adiposity indices. Also, blood pressure was measured twice, after a 10-minute seated rest. Adiposity indices were compared as to their sensitivity and specificity to predict hypertension. This study complies with the Helsinki Declaration and the procedures were approved by the Institution Review Board.

### 2.2. Sample

A total of six hundred and seventy-six people volunteered to participate. Eligibility criteria for the present analysis were as follows: to be aged between 20 and 60 years old and to live in the community for at least one year. All subjects who did not meet these two eligibility criteria were excluded from the present analysis. Thus, this study reports data from the five hundred and eighteen adult men and women (40.9 ± 10.5 years; 1.62 ± .09 m; 72.3 ± 15.6 kg) who were eligible and completed blood pressure and anthropometric measures. All participants were informed about the study procedures and voluntarily signed an informed consent form.

### 2.3. Blood Pressure and Hypertension

It is known that office measurements of blood pressure (BP) do not reflect diurnal variation and nocturnal BP levels [12]. Thus, hypertension was determined in the present study by analyzing participant's answers in the medical history questionnaire. All subjects were oriented to answer the questionnaire based on clinical examination performed prior to the present investigation. Therefore, it was assumed that all subjects that reported hypertension did so based on previous diagnostic made by a cardiologist through accurate method, such as 24 h ambulatory BP monitoring. Nevertheless, BP of each volunteer was measured twice by trained technicians after a 10-minute seated rest. Measurements were taken by auscultation using a mercury sphygmomanometer. Systolic BP and diastolic BP were defined as the points of the appearance and disappearance of Korotkoff sounds, respectively. Mean of the two measurements was calculated and recorded as the BP value. These values were used as secondary data to assess hypertension adopting systolic and diastolic BP cutoffs of 140 mmHg and 90 mmHg, respectively [13]. In addition, prevalence of diabetes* mellitus*, smoking status, alcohol consumption, and physical activity status were also assessed by questionnaire.

### 2.4. Anthropometry

Total body mass was measured in a digital scale to the nearest 0.50 g (OMROM HBF 510, OMRON Healthcare Inc., Lake Forest, IL). Height was measure to the nearest 0.1 cm using a portable stadiometer (Sanny®, São Bernardo do Campo, SP, Brazil). Waist circumference was assessed at the level of umbilicus, and hip circumference was determined at the level of the maximum extension of the buttocks posteriorly in a horizontal plane. Thus, WHR and WSR ratios were calculated as waist divided by hip and height (in centimeters), respectively. BMI was calculated as weight divided by height squared (kg/m^2^) and BAI was calculated according to the following equation [9]:

(1) $$\begin{array}{c} BAI=\left[\;\frac{\left(hip\;\;circumference\right)}{\left({\left(height\right)}^{1.5}\right)}-18\;\right]. \end{array}$$

Finally, the conicity index was determined according to the following equation [14]:

(2) Conicity  index=waist  circumference m0.109√total  body  mass kg/height m.

### 2.5. Statistical Analysis

Descriptive data are expressed as mean and standard deviation unless otherwise noted. The normal distribution of data was examined using the Kolmogorov-Smirnov test. Comparisons between men and women were carried out by independent* t*-test and chi-square test for continuous and categorical variables, respectively. The cut-off points for each adiposity index to predict hypertension were obtained using Receiver Operating Characteristic (ROC) curve analyses. Thus, areas under the ROC curves and the confidence intervals (CI: 95%) were used to compare the ability of each adiposity index to predict hypertension. Therefore, odds ratios (CI: 95%) for the presence of hypertension considering the identified cut-off points for each adiposity index were calculated. Moreover, Mantel-Haenszel provided common odds ratios adjustments for age, smoking, physical activity, diabetes, and alcohol consumption. The significance level was set at* P* ≤ .05, and all analyses were conducted using the Statistical Package for Social Sciences (SPSS 20.0).

## 3. Results

The overall prevalence of hypertension was 24.3% (95% CI: 20.3–28.0). Rates by gender were 23.9% (95% CI: 17.6–30.1) and 24.6% (95% CI: 19.9–29.2) for men and women, respectively, with no differences between groups (*X*^2^ = .031;* P* = .861). Based on BP values, overall prevalence was slightly lower (20.8%; 95% CI: 17.2–24.5) with no differences between genders (23.3% and 19.6% for men and women, respectively;* X*^2^ = .966* P* = .326). Overall prevalence of diabetes* mellitus* was 4.4% (95% CI: 2.9–6.2). Rates by gender were 4.5% and 4.4% for men and women, respectively, with no difference between groups (*X*^2^ = .007;* P* = .933). In addition, there was no difference between genders with regard to smoking status (8.0% and 6.4% for men and women, respectively;* X*^2^ = .416;* P* = .519). However, chi-square test showed a significant difference between gender groups regarding the prevalence of alcohol consumption (42.0% and 24.3% for men and women, respectively;* X*^2^ = 17.384;* P* < .001) and physically active status (74.4% and 52.9% for men and women, respectively;* X*^2^ = 22.441;* P* < .001). None of the participants reported heart disease.

Table 1 shows descriptive values regarding genders and its comparisons. Of note, no differences were observed for age, BMI, hip circumference, and WSR ratio between gender groups.

**Figure 1:**
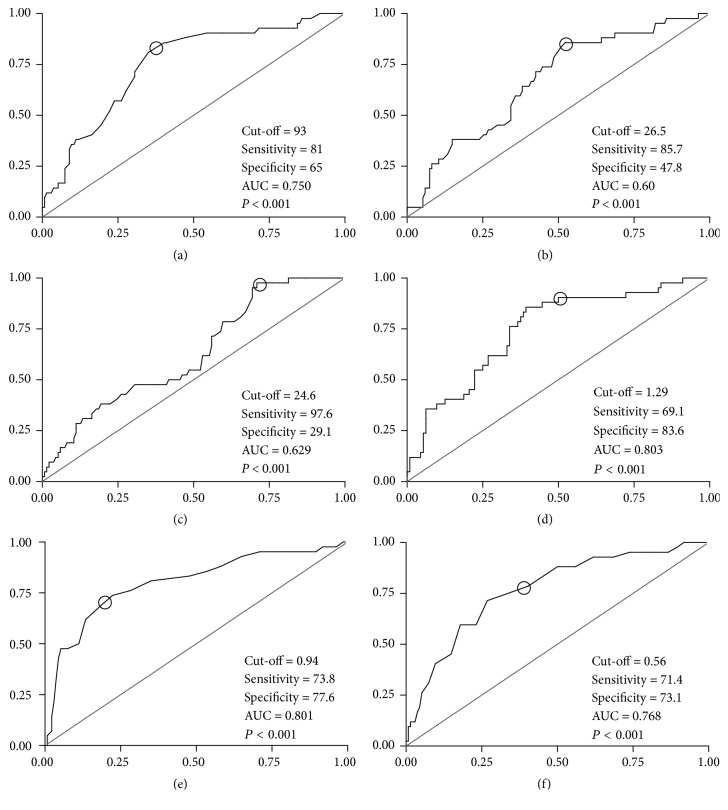
Receiver Operating Characteristic curve according to sensitivity (x-axis) and specificity (y-axis) of (a) waist circumference; (b) body mass index; (c) body adiposity index; (d) conicity index; (e) waist-to-hip ratio; (f) waist-to-stature ratio, in men. AUC: area under the curve.

**Figure 2:**
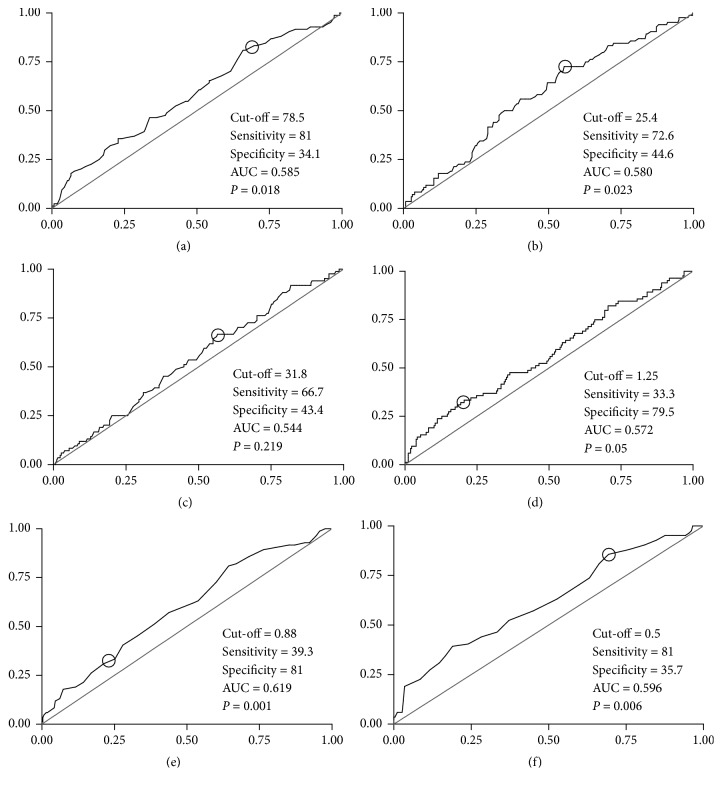
Receiver Operating Characteristic curve according to sensitivity (x-axis) and specificity (y-axis) of (a) waist circumference; (b) body mass index; (c) body adiposity index; (d) conicity index; (e) waist-to-hip ratio; (f) waist-to-stature ratio, in women. AUC: area under the curve.

ROC curves for each adiposity index according to sensitivity and specificity in the diagnosis of hypertension for men and women are presented in Figures 1 and 2, respectively. Regarding men's difference between areas under the ROC curves, pairwise comparisons highlighted C index, WHR, and WSR as higher than BMI (*P* < .001), WC (*P* < .05), and BAI (*P* < .001). Furthermore, BAI showed highest sensitivity (97.6) to predict HAS, meanwhile C index presented highest specificity (83.6). Moreover, both WHR and WSR were considered as more balanced indices regarding their sensitivity (73.8 and 71.4, respectively) and specificity (77.6 and 73.1, respectively). In women, WHR and WSR presented areas under the ROC curves higher than C index (*P* = .007) and BAI (*P* = .03), respectively.

Table 2 shows the odds ratios and confidence intervals for the presence of hypertension regarding each adiposity index above the proposed cut-off values. All adiposity indices regarding both genders showed significant odds ratios, except the BAI (odds ratio: 1.534; CI: 0.916–2.571) for women. Moreover, after adjustments for age, smoking, physical activity, diabetes, and alcohol consumption, the odds ratios for BAI in men was no longer statistically significant (odds ratio: 10.489; CI: 0.788–139.597). Interestingly, in women, there was no significant odds ratio after the adjustments.

## 4. Discussion

The purpose of this cross-sectional investigation was to analyze and compare several anthropometric indices in the prediction of hypertension among Brazilian adults. In general, the adiposity indices were more sensitive to predict hypertension in men. Of note, both WC and WSR presented values with the highest balance of sensitivity and specificity in the prediction of hypertension in men. Moreover, WC and WSR presented the highest sensitivity, while WHR presented the highest specificity to predict hypertension in women. Also, the prevalence of hypertension was 24.3%, without differences between genders. Finally, after adjustments for age, smoking, physical activity, diabetes, and alcohol consumption, all adiposity indices, except for BAI, showed significant odds ratios in men. On the other hand, there was no significant odds ratio in women after the adjustments.

Worldwide cut-off recommendations for BMI, WC, and WHR are 30 kg/m^2^ (both genders), 102 and 88 cm (men and women), and 0.90 and 0.85 cm (men and women), respectively [15]. Using these recommended cut-off points of BMI and WC to define obesity, a previous study [7] in a Brazilian sample (*n* = 592, both genders) observed hazard ratios adjusted for age and blood pressure of 1.08 (0.52–2.24,* P* = .82) and 1.74 (0.93–3.26,* P* = .08) for BMI in men and women, respectively. Regarding WC, hazard ratios were 1.78 (0.76–4.09,* P* = .18) for men and 1.72 (1.09–2.73,* P* = .02) for women. As only WC for women presented a significant ability to predict hypertension, the authors of that study concluded that the risk for hypertension may be better defined by higher WC than higher BMI. In general, the results of the present study presented lower cut-off values when compared to the aforementioned. For instance, WC cut-off points to predict hypertension were 9.0 cm lower in men and 9.5 cm lower in women than the general cut-off recommended by the World Health Organization. This is an important finding that highlights the need for continuously educating people about hypertension risk factors in the sample of this study.

Moreover, there is a gap for the cut-off points of WSR, BAI, and C index. Despite this gap, a recent investigation observed that WSR is more strongly associated with hypertension than BMI, WC, and WHR in men and women from India and Pakistan [8]. In line with this, a previous research analyzing a large sample of Chinese men and women (*n* = 2895; 25–74 years old) observed that WSR was the best index in predicting elevated systolic and diastolic BP in women when compared to BMI, WC, and WHR (WSR likelihood ratios in that study were 3.05 and 3.38 for systolic and diastolic BP, respectively) [10]. In addition, in a large sample (*n* = 3971, both genders) of Arab adults, Al-Daghri and colleagues [16] found that WSR was the most sensitive index, but rather modest, in determining hypertension (AUC 0.66,* P* < .001). In the present research, these values were even higher (see Figures 1 and 2). However, as Al-Daghri et al. did not present sensitivity and specificity for genders separately, further comparison with the present study is limited. In general, the present investigation does not totally corroborate those previous data, once none of the indices presented significant odds ratios to predict hypertension in women after the adjustments, while all of them (except BAI) presented significant odds ratio to predict hypertension in men.

Regarding newer adiposity indices such as BAI, further investigations are clearly warranted, as previous research came up with conflicting results. For instance, a recent report identified BAI to be a useful index to predict hypertension in adult obese women from India (*n* = 131; odds ratio = 3.28, CI: 1.95–5.51,* P* = .001) [17]. In the present study, BAI did not present significant odds ratio in women, nor in men, after adjusting for age, smoking, physical activity, diabetes, and alcohol consumption. Indeed, there is evidence that BAI may be an inferior index to predict body fatness and cardiovascular risk factors (i.e., including systolic and diastolic blood pressure) compared to BMI and WC [18, 19]. In the Bogalusa Heart Study [19], for example, the authors showed that the correlation between BAI and a sum of cardiovascular risk factors (lipids, fasting insulin and glucose, and blood pressure) in 2367 men and women (18–49 years old) is significantly weaker when compared with BMI and WC (*r* = .49, .58, and .61, respectively).

Interestingly, in the present study, adiposity indices were more sensitive to predict hypertension in men. Moreover, indices that consider abdominal obesity (i.e., WC and WSR) presented high balance of sensitivity and specificity to predict hypertension in men, but not in women. This finding can be explained by the differences of fat distribution between genders. Indeed, men usually present more fat in the central region of the body, and abdominal fat accumulation (i.e., android pattern) is associated with higher risk for cardiovascular diseases, independent of total body mass or BMI; otherwise, fat accumulation in the subcutaneous or appendicular skeleton (i.e., usually presented by women) is considered to be less harmful [20, 21]. These findings corroborate previous findings and the guidelines of the World Health Organization which states that measures of abdominal obesity are better than BMI as predictors of hypertension and other cardiovascular risk factors [15, 22]. In light of the present results allied with previous investigations regarding differences in gender, the “need to develop sex specific cut-off values appropriate for different populations” has been stated [15]; hence there is no consensus of these values in the literature regarding hypertension in adults. Additionally, all cutoffs in the present study were adjusted according to its sensitivity and specificity to predict hypertension in a specific population. Thus, these findings contribute to the management of hypertension in the studied population.

Once office measurements of BP may not reflect the true BP levels (i.e., they do not reflect diurnal variation and nocturnal BP levels) [12], our first choice to determine hypertension was based on participant's answers to the medical history questionnaire. Consistent with previous reports [5], the prevalence of hypertension found in this investigation (24.3%, 95% CI: 20.3–28.0) is similar to that of the overall Brazilian population of big urban centers (around 24.8%) [5]. These values are higher than the prevalence found recently in Canada (19.5%), but lower when compared to other developed countries such as the United States of America (29.1%) and England (30.0%) [2]. When compared to other middle-income countries such as México (44.6%), China (42.7%), and India (37.8%), the prevalence of hypertension among the present sample was found to be lower. Some lifestyle habits may be related to this high prevalence of hypertension in developing countries. For instance, in Brazil, daily consumption of sodium is more than twice (4.7 g per 2000 kcal) the recommended levels of intake according to the World Health Organization [23]. In addition, despite the fact that around 60% of the sample of this study reported being physically active (data not shown), sedentary lifestyle in leisure-time is high (around 65% of the people) among Brazilians [5, 24]. Of note, incidence of hypertension and other cardiovascular and metabolic diseases is growing worldwide. Once hypertension is a common condition that may lead to myocardial infarction, stroke, renal failure, and death if not detected early and treated appropriately [4], public health strategies to overcome this disease are imperious.

The present study has several strengths and limitations. Representative sample and novelty of the results are strengths. We opted to evaluate body adiposity using easy-access and low-cost clinical indices in a large sample instead of gold standard that would certainly demand higher costs and lower external validity. The study is limited by its cross-sectional design, which precludes cause-effect inferences. Thus, although the present report adds information to the literature, longitudinal studies are recommended to establish the temporal influence of predicting ability of clinical adiposity indices and hypertension in adults.

## 5. Conclusions

Consistent with previous reports, various adiposity indices are associated with hypertension in Brazilian adults with different degrees. We observed that indices which consider abdominal adiposity such as WC, WHR, and WSR have a stronger relationship to hypertension compared to others. Of note, adiposity indices were more sensitive to predict hypertension in men and this is probably related to the differences of fat distribution between genders. Given that WC, WHR, and WSR are accurate, are simple to measure, and have minimal cost, they should be routinely employed in the assessment of Brazilian adults at high risk for hypertension.

## Figures and Tables

**Table 1:** Descriptive values and comparison between genders (mean ± SD).

	Men	Women	*P*
*n*	176	342	
Age (years)	41.3 ± 10.5	40.7 ± 10.5	.514
Height (m)	1.71 ± 0.1	1.6 ± 0.1	**.001**
Body weight (kg)	81.0 ± 14.7	67.8 ± 14.2	**.001**
Body mass index (kg/m^2^)	27.7 ± 4.3	27.2 ± 5.1	.222
Waist circumference (cm)	93.2 ± 11.9	85.0 ± 11.5	**.001**
Hip circumference (cm)	101.0 ± 8.3	102.1 ± 10.4	.203
Waist-to-hip ratio	0.92 ± 0.1	0.83 ± 0.1	**.001**
Waist-to-stature ratio	0.55 ± 0.1	0.54 ± 0.1	.287
Body adiposity index (%)	27.3 ± 3.7	33.6 ± 5.3	**.001**
Conicity index (AU)	1.2 ± 0.1	1.2 ± 0.1	**.001**
Systolic BP (mmHg)	124.1 ± 13.2	120.3 ± 13.7	**.003**
Diastolic BP (mmHg)	79.9 ± 10.4	77.7 ± 9.8	**.016**

*Note.* AU: Arbitrary units. BP: blood pressure.

**Table 2:** Odds ratios (OR, confidence interval: 95%) for hypertension.

	Men (*n* = 176)		Women (*n* = 342)	
Indices	OR (CI = 95%)	Adjusted OR	OR (CI = 95%)	Adjusted OR
WC	7.87 (3.37–18.37)	4.29 (1.51–12.22)	2.20 (1.21–4.02)	1.40 (0.67–2.96)
BMI	5.17 (2.04–13.08)	6.33 (1.61–24.84)	2.07 (1.21–3.54)	1.84 (0.94–3.61)
BAI	16.22 (2.16–122.22)	10.49 (0.79–139.60)	1.53 (0.92–2.57)	1.50 (0.89–2.56)
C Index	10.18 (4.63–22.38)	6.60 (1.90–22.95)	1.76 (1.03–3.03)	1.35 (0.68–2.68)
WHR	7.97 (3.62–17.54)	3.34 (1.30–8.58)	2.51 (1.48–4.26)	1.95 (0.99–3.84)
WSR	6.66 (3.04–14.14)	4.20 (1.57–11.21)	2.20 (1.21–4.02)	1.48 (0.69–3.18)

*Note. *Odds ratio adjusted for age, smoking, physical activity, diabetes, and alcohol consumption.

## References

[1] Sociedade Brasileira de Cardiologia (2010). VI Diretrizes Brasileiras de Hipertensão. *Arquivos Brasileiros de Cardiologia*.

[2] Joffres M., Falaschetti E., Gillespie C. (2013). Hypertension prevalence, awareness, treatment and control in national surveys from England, the USA and Canada, and correlation with stroke and ischaemic heart disease mortality: a cross-sectional study. *BMJ Open*.

[3] Lloyd-Sherlock P., Beard J., Minicuci N., Ebrahim S., Chatterji S. (2014). Hypertension among older adults in low and middle-income countries: prevalence, awareness and control. *International Journal of Epidemiology*.

[4] James P. A., Oparil S., Carter B. L. (2013). 2014 Evidence-based guideline for the management of high blood pressure in adults. Report from the panel members appoints to the Eighth Joint National Commitee (JNC 8). *Journal of the American Medical Association*.

[5] Brasil. Ministério da Saúde. Secretaria de Vigilância em Saúde. Departamento de Vigilância de Doenças e Agravos não Transmissíveis e Promoção da Saúde Vigitel Brasil 2014: vigilância de fatores de risco e proteção para doenças crônicas por inquérito telefônico., Ministério da Saúde, Brasília, DF.

[6] Cox B. D., Whichelow M. J., Prevost A. T. (1998). The development of cardiovascular disease in relation to anthropometric indices and hypertension in British adults. *International Journal of Obesity*.

[7] Gus M., Fuchs S. C., Moreira L. B. (2004). Association between different measurements of obesity and the incidence of hypertension. *American Journal of Hypertension*.

[8] Patel S. A., Deepa M., Shivashankar R. (2017). Comparison of multiple obesity indices for cardiovascular disease risk classification in South Asian adults: the CARRS study. *PLoS ONE*.

[9] Bergman R. N., Stefanovski D., Buchanan T. A. (2011). A better index of body adiposity. *Obesity*.

[10] Ho S.-Y., Lam T.-H., Janus E. D. (2003). Waist to stature ratio is more strongly associated with cardiovascular risk factors than other simple anthropometric indices. *Annals of Epidemiology*.

[11] Associação Brasileira para o Estudo da Obesidade e da Síndrome Metabólica (Abeso) (2009). *Diretrizes Brasileiras de Obesidade 2009-2010*.

[12] Grossman E. (2013). Ambulatory blood pressure monitoring in the diagnosis and management of hypertension. *Diabetes Care*.

[13] Malachias M. V. B., Souza W. K. S. B., Plavnik F. L., Rodrigues C. I. S. (2016). 7^a^ diretriz brasileira de hipertens*π*o arterial. *Arquivos Brasileiros de Cardiologia*.

[14] Taylor R. W., Jones I. E., Williams S. M., Goulding A. (2000). Evaluation of waist circumference, waist-to-hip ratio, and the conicity index as screening tools for high trunk fat mass, as measured by dual-energy X-ray absorptiometry, in children aged 3-19 y. *American Journal of Clinical Nutrition*.

[15] Nishida C., Ko G. T., Kumanyika S. (2010). Body fat distribution and noncommunicable diseases in populations: overview of the 2008 WHO Expert Consultation on Waist Circumference and Waist–Hip Ratio. *European Journal of Clinical Nutrition*.

[16] Al-Daghri N. M., Al-Attas O. S., Wani K. (2015). Sensitivity of various adiposity indices in identifying cardiometabolic diseases in Arab adults. *Cardiovascular Diabetology*.

[17] Gupta S., Kapoor S. (2014). Body adiposity index: its relevance and validity in assessing body fatness of adults. *ISRN Obesity*.

[18] Freedman D. S., Thornton J. C., Pi-Sunyer F. X. (2012). The body adiposity index (Hip Circumference ÷ Height^1.5^) is not a more accurate measure of adiposity than is BMI, waist circumference, or hip circumference. *Obesity*.

[19] Freedman D. S., Blanck H. M., Dietz W. H., DasMahapatra P., Srinivasan S. R., Berenson G. S. (2013). Is the body adiposity index (hip circumference/height(1.5)) more strongly related to skinfold thicknesses and risk factor levels than is BMI? The bogalusa heart study. *British Journal of Nutrition*.

[20] Cameron A. J., Magliano D. J., Söderberg S. (2013). A systematic review of the impact of including both waist and hip circumference in risk models for cardiovascular diseases, diabetes and mortality. *Obesity Reviews*.

[21] Dutra M. T., Gadelha A. B., Nóbrega O. T., Lima R. M. (2017). Body adiposity index, but not visceral adiposity index, correlates with inflammatory markers in sarcopenic obese elderly women. *Experimental Aging Research*.

[22] Fuchs F. D., Gus M., Moreira L. B. (2005). Anthropometrie indices and the incidence of hypertension: a comparative analysis. *Obesity Research*.

[23] Sarno F., Claro R. M., Levy R. B., Bandoni D. H., Monteiro C. A. (2013). Estimated sodium intake for the Brazilian population, 2008-2009. *Revista de Saúde Pública*.

[24] Andrade A. C. D. S., Peixoto S. V., Friche A. A. D. L. (2015). Social context of neighborhood and socioeconomic status on leisure-time physical activity in a Brazilian urban center: the BH health study. *Cadernos de Saúde Pública*.

